# Beyond the 90-90-90: refocusing HIV prevention as part of the global HIV response

**DOI:** 10.7448/IAS.19.1.21348

**Published:** 2016-12-19

**Authors:** Rachel Baggaley, Shona Dalal, Cheryl Johnson, Virginia Macdonald, Ioannis Mameletzis, Michelle Rodolph, Carmen Figueroa, Julia Samuelson, Annette Verster, Meg Doherty, Gottfried Hirnschall

**Affiliations:** HIV Department, World Health Organization, Geneva, Switzerland

**Keywords:** HIV prevention, antiretroviral therapy, voluntary medical male circumcision, PrEP, key populations

## Abstract

**Introduction:**

The remarkable expansion in availability of antiretroviral therapy (ART) over the past two decades has transformed HIV infection into a manageable chronic condition. People with HIV infection now live long and healthy lives on treatment that is simpler, safer and cheaper. According to UNAIDS estimates, the global coverage of ART reached 46% in 2015, resulting in a 26% decrease in annual HIV-related deaths since 2010. Such success has positioned treatment access at the centre of the global HIV response as a way to prevent mortality, morbidity and HIV transmission through a “Treat All” approach. Continuing expansion of treatment is needed to further reduce HIV-related mortality. This progress with treatment, however, masks a stagnation in the estimated annual number of new HIV infections. Continuing levels of HIV incidence despite treatment scale-up stem from several factors, which should be addressed in order to prevent new infections and decrease the numbers of people requiring treatment in the future.

**Discussion:**

ART can only reach those already diagnosed, and although it is unclear what proportion of new infections occur during acute and early infection prior to treatment initiation, phylogenetic studies suggest that it might be substantial. Thus, better testing approaches to reach the 40% of people with undiagnosed HIV infection as early as possible are critical. New approaches to reach men, young people and key populations, where HIV risk is highest and HIV prevention, testing and treatment coverage is lowest, are also needed. Overall coverage of effective prevention interventions remains low, enabling HIV transmission to occur, or time is required to show population-level effects. For example, the full impact of the medical male circumcision intervention will be seen once a larger proportion of men in age cohorts with high incidence are circumcised. Finally, strategically focused pre-exposure prophylaxis interventions have the potential to prevent HIV acquisition among populations at substantial risk, averting treatment costs in coming years.

**Conclusions:**

The United Nations (UN) targets to end AIDS include the “90-90-90” targets for HIV diagnosis, treatment and viral suppression. While 90-90-90 has been widely emphasized and adopted by countries and international funders, the focus thus far has largely been on increasing access to ART – the second “90.” A similar emphasis on achieving UN HIV prevention targets and adequate funding for meeting these is essential, alongside treatment, in order to reduce population-level incidence and change the trajectory of the HIV epidemic over the long term.

## Introduction

The HIV response and the outlook for people with HIV looked very different at the 2016 International AIDS conference in Durban, South Africa, than it did in 2000 when the 13th Conference was also held in Durban. Approximately 12,000 people attended the 2000 conference, and Jeffrey Sachs reinforced the call by the Group of Eight and the World Health Organization (WHO) for a global fund to fight AIDS, which was established just over a year later. Nelson Mandela gave the closing address, where he stated “this is, as I understand it, a gathering of human beings concerned about turning around one of the greatest threats humankind has faced.” In the intervening 16 years, a remarkable turnaround has indeed occurred. HIV is now, as a result of the scale-up in antiretroviral therapy (ART), a treatable infection, with millions of people with HIV living long and healthy lives. The Global Fund to Fight AIDS, Tuberculosis and Malaria has provided more than $15 billion and the United States President's Emergency Plan for AIDS Relief (PEPFAR) has provided more than $50 billion in HIV funding to support this. The prices of antiretroviral drugs have reduced substantially and formulations are simpler and safer, and the confirmation of the clinical benefits of early treatment is recognized with new treatment recommendations to “Treat All” being widely adopted as policy [1]. Decentralizing service delivery and task sharing have also contributed to increasing ART coverage, particularly across sub-Saharan Africa. The latest UNAIDS report estimates that the global coverage of ART reached 46% in 2015, contributing to a 26% decrease in annual HIV-related deaths since 2010 [2]. Such success has positioned treatment access at the centre of the global HIV response as a way to prevent mortality and morbidity and ongoing HIV transmission to infants of positive mothers, or to sexual and drug-injecting partners. To date, this message has been well accepted and further scale-up of ART coverage is seen as the keystone to achieve the United Nations (UN) 90-90-90 targets to diagnose 90% of all people with HIV, to treat 90% of those diagnosed and for 90% of people treated to be virally suppressed [3]. Although this focus and progress on the second of the three “90s” has been critical to reducing morbidity and mortality, efforts in improving the diagnosis of people with HIV who are not aware of their status, and in achieving viral suppression among those on treatment, need further attention.

Despite these achievements, earlier ART and support for better retention on ART are needed to reduce annual mortality rates from HIV further, and UNAIDS reports that there has not been a significant decrease in estimated new infections since 2010. With approximately 2 million (1.7–2.2 million) new infections in 2015, it is now estimated that there are 36.7 million (34.0 million −39.8 million) people with HIV globally [2]. Furthermore, not only are rates of new adult infections static, but some regions also continue to experience increases in new infections. In Eastern Europe and Central Asia, where HIV is primarily concentrated in key populations, there was an estimated 57% increase in annual new infections between 2010 and 2015 [2].

## Discussion

### The limits of treatment as prevention

Although it was anticipated [4], a reduction in new HIV infections because of the global increase in ART coverage has not yet been demonstrated and needs to be understood. At an individual level, two studies found ART to be highly effective in preventing HIV transmission between partners [5,6], reporting >96% effectiveness among serodiscordant heterosexual couples [5] and nearly 100% effectiveness among virally suppressed serodiscordant heterosexual and male same-sex partners [6]. This heralded optimism that the >90% preventive effect of ART, which is widely used (or approximations to it) in modelling, was forecast to result in the rapid translation of 90% reduction in new HIV infections at a population level by 2030 [3].

However, large reductions in incidence have not been seen. Most recently, a large trial assessing the impact of universal “test and treat” on HIV incidence found no reduction in incidence following the intervention [7]. The increase in ART coverage over the past five years in East and Southern Africa has been remarkable, with an estimated 30% increase in ART coverage, from 24% (22–26%) in 2010 to 54% (50–58%) in 2015. Although this scale-up has resulted in a 36% decrease in annual HIV-related deaths in the region, during the same period, there was no significant decline in estimated new adult HIV infections [2]. This may be either because it is too early to see a population-level effect of ART scale-up or because ART coverage will actually need to be much higher and sustained, and be of sufficient quality, with achievement of viral suppression, in order to achieve a substantial reduction in new infections.

### Lack of impact of treatment scale-up on HIV incidence stems from several factors

First, even under the most optimistic scenarios, it is currently not possible to identify and treat people early enough after their infection to prevent further HIV transmission. A continuing concern is the estimated 40% of all people with HIV who remain undiagnosed, [8] and are therefore unlinked to prevention, treatment and care services and continue to be at risk of transmitting HIV vertically (mother-to-child transmission) or to sexual and drug-injecting partners. Better testing approaches which can reach those with undiagnosed HIV infection, as early in their infection as possible, need support and funding. These could include medical indicator condition-guided HIV testing, HIV self-testing and routinely offering assisted partner notification services to all persons diagnosed with HIV infection during their interactions with health services.

Without developing and implementing new approaches to reach men, young people and key populations where HIV risk is highest and HIV testing coverage is lowest, the benefits of testing and linkage to prevention, treatment and care will continue to be unrealized [9]. This is reflected in the fact that although ART coverage has increased substantially over the last decade, the average CD4 cell count at the start of treatment has only increased modestly [10,11]; many people still only start ART when they are at an advanced stage of immunosuppression, implying many years between infection and initiation of ART.

Even for the relatively limited number of people diagnosed early following infection, it may not have been early enough to initiate treatment, achieve viral suppression and prevent transmission. It is unclear what proportion of new infections (transmissions) occur during acute and early phases of HIV infection (before people can be feasibly diagnosed and started on ART). However, it is clear that despite high ART coverage in many high-income settings, HIV incidence is not falling. Phylogenetic analysis of viral sequences suggests that in the Netherlands 70% of new infections arise from undiagnosed people, and 22% during acute and recent HIV infection [12]. A phylogenetic transmission analysis with matched epidemiological and clinical data among men who have sex with men (MSM) in the United Kingdom shows a similar pattern with recent infection contributing disproportionately to secondary HIV transmission [13]. In Vancouver, Canada, it was reported that high ART coverage had resulted in decreases in community viral load and new HIV diagnoses [14]; however, the role of increased coverage of comprehensive harm reduction for people who inject drugs in driving these trends is unclear [15]. Government HIV surveillance reported little change in new diagnoses among MSM in British Columbia, Canada [16].

Second, high levels of treatment coverage are not the same as high levels of virological suppression as emphasized by the third 90 UN target for testing and treatment. Suboptimal adherence and high rates of loss to follow-up continue to influence the overall effectiveness of ART. A recent analysis of rates of virological suppression in low- and middle-income settings found that when loss to follow-up was taken into consideration, only around 62% of patients starting ART were virologically suppressed four years after starting ART [17] and in the United States only two-thirds of people in HIV care had durable viral suppression during a two-year period [18].

Third, models predicting the impact of ART on HIV incidence at the population level are also predicated on high levels of coverage of prevention interventions [19]. However, coverage of key prevention interventions remains too low and may take time to take effect. For example, the population-level impact of voluntary medical male circumcision (VMMC), an effective one-time intervention, is likely to only be fully realized in coming years once a larger proportion of adolescent boys and men are circumcised in age cohorts with high incidence or prior to reaching sexual debut. Similarly, there is a gap between need and availability of condoms in sub-Saharan Africa [8].

Fourth, female-controlled prevention options have been few thus far, although pre-exposure prophylaxis (PrEP) shows promise for both women and men. Pairing increased treatment access with refocused interventions to prevent new infections among women will be needed to make the progress required to turn around the HIV epidemic.

Fifth, key populations, which have continued high HIV incidence, are still faced with social and legal barriers, which increase their risk of HIV exposure and acquisition and inhibit access to evidence-based HIV prevention and treatment services. For example, despite compelling evidence of the effectiveness of harm reduction interventions, countries remain reluctant to implement needle and syringe programmes (NSPs) and opioid substitution therapy, or to bring them to scale.

### Prevention is contextual and often perceived as “complicated, difficult and boring”

Interest, prioritization and funding for HIV prevention interventions and programmes from countries, donors and communities have been inadequate over the last decade. For example, in 2015, only 7% of the estimated funds required to reach enough people who inject drugs with evidence-based prevention services to have an impact on the epidemic was available from international donors [20].

Significant prevention gains were made in some countries in East and Southern Africa, such as Uganda and Zimbabwe, before ART became widely available; paradoxically, these gains have diminished over the past five years where ART coverage has increased. National surveys suggest that trends in indicators associated with higher HIV risk behaviours – early sexual debut, multiple sexual partners and unprotected sex – have increased compared with the declines reported in 2000–2010. Condom availability and funding has decreased in some settings, with countries recently reporting stock-outs. As a large new generation of adolescents is emerging, they often have less knowledge about HIV prevention than the cohort before them. Yet, today, HIV prevention choices are more varied and effective than ever before.

Providing effective and acceptable prevention services across countries and populations is complex. Unlike ART, where recommendations are universal across populations and regions, what to offer and how to provide effective and acceptable prevention varies by population and epidemiological context [21]. Human behavioural factors also compound the successful adoption of prevention interventions, and often difficult legal and social considerations need to be addressed for effective service delivery. Furthermore, laws and policies such as mandatory testing and criminalization of HIV transmission inhibit HIV test uptake and treatment access. WHO does not support mandatory testing or laws and policies which criminalize HIV transmission or behaviours of key populations.

VMMC is a cost-effective, one-off, permanent, lifelong prevention intervention shown to decrease the risk of heterosexually acquired HIV in men by >60%. By the end of 2015, 14 countries in Africa have delivered a comprehensive HIV prevention package including circumcision to more than 12 million adolescent boys and men. The impact of VMMC on HIV incidence, although demonstrated in Uganda, will likely have been limited to date, but will increase over the next five years as the circumcised adolescent boys and younger men become sexually active and reach ages where incidence has been greatest to date.

An impressive body of evidence confirms that PrEP is highly effective in preventing HIV acquisition [22] and can be cost-effective if provided for people at substantial HIV risk. PrEP programmes are starting to be prioritized for MSM in high- and middle-income countries, and a national programme providing PrEP for sex workers was launched in South Africa in June 2016. PrEP services need to be implemented well and focused strategically – a balanced approach to creating demand and delivering PrEP within existing services will be needed for maximum acceptability and impact. If implemented well, PrEP programmes will not only provide effective prevention for those at ongoing substantial risk of infection, but also reach people at high HIV risk with HIV testing services. This should increase the diagnoses of new HIV infections and facilitate linking people with HIV to ART services, as a feasible approach to identifying and treating people during acute and early HIV infections to reduce HIV transmission.

### Prioritizing key populations

The UNAIDS report also indicates that with the exception of sub-Saharan Africa, more than 90% of new HIV infections in other parts of the world in 2014 were among people from key populations (MSM, sex workers, people who inject drugs, transgender people, and people in prisons and other closed settings) and their sexual partners. In sub-Saharan Africa, these populations accounted for more than 20% of new HIV infections. Key populations are still not being reached at scale or with effective HIV prevention, testing and treatment services despite having the highest rates of HIV incidence and prevalence.

Some countries continue to prioritize HIV prevention efforts among the general population even where the prevalence is very low. Social and cultural unease and legal barriers often get in the way of accepting the public health impact of interventions and result in a failure to provide HIV services to key populations in many countries. Structural and social determinants of risk, such as violence and criminalization, can be addressed through successful prevention programmes focusing on key populations, although such measures are perceived to be difficult. However, although the current social and legal environment makes it often unpopular and difficult to recognize and support services for key populations, the HIV response should be driven by epidemiologic evidence in all countries and should prioritize high-impact interventions for populations at increased risk of infection. There is the potential for new interventions whose impact outside of clinical research has yet to be realized. PrEP is one example where its implementation at scale may have an important place in HIV prevention efforts.

People who live in prisons and other closed settings are often ignored in the response, although they have increased HIV, tuberculosis and hepatitis prevalence and high rates of risk behaviours, including injecting drug use and unprotected sex while incarcerated. Risk behaviours may continue after their release from prison and return to their communities. There is also a significant overlap of networks between different key population groups, with incarceration rates high among people who inject drugs and sex workers. Investment in HIV prevention programmes in the community can be made redundant by a complete lack of services within prisons: while community-based NSPs are available in 90 countries, only eight countries provide prison NSPs. Condom programmes, available almost universally in community settings, are only provided in prisons in 28 countries [23].

Strategic information is being used in a wide range of countries to refocus their HIV resources, to reach key populations and invest in high-impact interventions. A number of development agencies are providing incentives for countries to make more strategic choices in investing their HIV resources. For example, PEPFAR has announced the establishment of a new investment fund for key populations, and the Global Fund highlights the importance of addressing key populations in the development of concept notes for funding. Identifying and scaling up effective and comprehensive prevention and treatment services for key populations will contribute significantly towards reducing the numbers of new HIV infections over time.

## Conclusions

The UN 90-90-90 targets for HIV diagnosis, treatment and viral suppression have been widely promoted and adopted by countries and international funders. While the scale-up of treatment has contributed to saving millions of lives and reducing major illnesses, there has also been an increasing expectation that treatment scale-up will have an impact on HIV incidence at a population level. At a global level, even in cities such as Amsterdam, London and Paris, where 90-90-90 targets have been reached [24], this has not yet been shown. These data highlight the need to continue to scale up access to HIV treatment, as soon as possible after infection, and in particular for key populations. The promise of “treatment-as-prevention” is predicated on high coverage of testing and prevention interventions and high ART adherence and retention. A similar emphasis and push for the UN prevention targets and funding for prevention, with specific prevention packages for different populations, should be embraced alongside the ART scale-up in order to reduce population-level incidence and change the trajectory of the HIV epidemic over the long term.
